# Recent changes in trends of mortality from cervical cancer in Southeastern Brazil

**DOI:** 10.11606/s1518-8787.2023057004709

**Published:** 2023-08-28

**Authors:** Carolina Terra de Moraes Luizaga, Beatriz Cordeiro Jardim, Victor Wünsch, José Eluf, Gulnar Azevedo e Silva

**Affiliations:** I Fundação Oncocentro de São Paulo São Paulo SP Brasil Fundação Oncocentro de São Paulo. São Paulo, SP, Brasil; II Universidade do Estado do Rio de Janeiro Instituto de Medicina Social Hesio Cordeiro Rio de Janeiro RJ Brasil Universidade do Estado do Rio de Janeiro. Instituto de Medicina Social Hesio Cordeiro. Rio de Janeiro, RJ, Brasil; III nstituto Nacional de Câncer Rio de Janeiro RJ Brasil Instituto Nacional de Câncer. Rio de Janeiro, RJ, Brasil; IV Universidade de São Paulo Faculdade de Saúde Pública São Paulo SP Brasil Universidade de São Paulo. Faculdade de Saúde Pública. São Paulo, SP, Brasil; V Universidade de São Paulo Faculdade de Medicina São Paulo SP Brasil Universidade de São Paulo. Faculdade de Medicina. São Paulo, SP, Brasil

**Keywords:** Uterine Cervical Neoplasms, Mortality Registries, Spatio-Temporal Analysis, Time Series Studies, Brazil

## Abstract

**OBJECTIVE:**

To analyze the trends of cervical cancer mortality in Brazilian Southeastern states, and to compare them to Brazil and other regions between 1980 and 2020.

**METHODS:**

Time series study based on data from the *Sistema de Informações de Mortalidade* (Brazilian Mortality Information System). Death data were corrected by proportional redistribution of deaths from ill-defined causes and cervical cancer of unspecified portion. Age-standardized and age-specific rates were calculated by screening target (25–39 years; 40–64 years) and non-target (65 years or older) age groups. Annual percentage changes (APC) were estimated by linear regression model with breakpoints. The coverage of Pap Smear exam in the Unified Health System (SUS) was evaluated between 2009 and 2020 according to age group and locality.

**RESULTS:**

There were increases in corrected mortality rates both in 1980 and in 2020 in all regions, with most evident increments at the beginning of the series. There was a decrease in mortality nationwide between 1980–2020; however, the state of São Paulo showed a discrete upward trend in 2014–2020 (APC=1.237; 95%CI 0.046–2.443). Noteworthy is the trend increment in the 25–39 year-old group in all study localities, being sharper in the Southeast region in 2013–2020 (APC=5.072; 95%CI 3.971–6.185). Screening coverage rates were highest in São Paulo and lowest in Rio de Janeiro, with a consistent decline from 2012 onwards at all ages.

**CONCLUSIONS:**

São Paulo is the first Brazilian state to show a reversal trend in mortality from cervical cancer. The changes in mortality patterns identified in this study point to the need for reorganization of the current screening program, which should be improved to ensure high coverage, quality, and adequate follow-up of all women with altered test results.

## INTRODUCTION

Cervical cancer is a disease necessarily caused by the persistent infection by high-risk human papillomavirus (HPV) types^1^ and, although potentially preventable, it remains a serious health issue in low- and middle-income countries^2^ . With large global variations in mortality rates, it is the leading cancer type related to death among women in 36 countries^2^ . In recent decades, significant reductions in mortality and incidence have occurred in countries that have implemented Pap Smear-based vaginal cytology^3^ screening programs, with better results found in those with organized screening^4^ .

Adversely, some countries that have seen significant declines in morbidity and mortality from organized Pap Smear-based screening have started to witness smaller declines, stability or even increases in mortality from the disease^5 - 6^ , which highlights the need for investments in more efficient strategies for organizing screening programs^7 - 8^ .

The first initiatives for early detection of cervical cancer in Brazil were isolated, within restricted populations and occurred in the late 1980s. It was only after 1998, with the development of a control program for this cancer by the Brazilian Ministry of Health (MoH), that cervical screening practices were structured on an opportunistic basis throughout the country. The current guidelines recommend the Pap Smear test for women aged 25–64 years^9^ . To monitor screening tests and diagnostic confirmation in the Unified Health System (SUS), the MoH implemented information systems called SISCOLO ( *Sistema de Informação do Câncer do Colo do Útero* , Cervical Cancer Information System) and SISCAN ( *Sistema de Informação do Câncer* , Cancer Information System).

In the state of São Paulo, data available from the State Health Secretariat indicate a decline in the coverage of Pap Smear exams performed at SUS as of 2010^10^ and problems in the follow-up of screened women^11^ , which may impact the trend of decreasing mortality in the medium and long term. The objective of this study is to analyze the trends of cervical cancer mortality in Brazilian Southeastern states, and to compare them to Brazil and other regions between 1980 and 2020.

## METHODS

Time-series study using data of deaths among women recorded in the Mortality Information System ( *Sistema de Informação sobre Mortalidade* , SIM) in the period 1980–2020. The data were obtained from the DATASUS^12^ website, with data for 2020 still preliminary. For 1980–1995 the ninth edition of the International Statistical Classification of Diseases, Injuries and Causes of Death (ICD-9) was used, and between 1996–2020 the tenth edition (ICD-10) was used.

Populations for each locality were obtained from tabulations in DATASUS^13^ according to: 1) 1980–2012 data from the Censuses (1980, 1991, 2000 and 2010), Count (1996) and Intercensal Projections (1981–2012); and, 2) 2013–2020 data from the Population Estimates Study.

Mortality data and populations were aggregated in 5-year intervals from 15 to 79 years old (0–14, 15–19, 20–24, 25–29, 30–34, 35–39, 40–44, 45–49, 50–54, 55–59, 60–64, 65–69, 70–74, 75–79 and 80 years or older) according to the Federation Unit for the Southeast, Brazil and Brazilian regions of residence. Deaths records without age information were proportionally distributed among the seven age groups according to the underlying cause of death, place of residence and year of death.

We corrected the information on the underlying cause of death applying the methodology proposed by Mathers et al.^14^ (2003) and adapted by Girianelli et al.^15^ (2014), which consists on proportionally redistributing 50% of deaths with an ill-defined underlying cause (ICD-9 780-799; ICD-10 R00-R99). This correction is identified in this study as Correction 1.

For deaths registered as from cervical cancer (ICD-9 180; ICD-10 C53), an additional correction (Correction 2) was performed, with redistribution of deaths classified as malignant neoplasm of uterus, part unspecified (ICD-9 179; ICD-10 C55), maintaining the proportion registered as deaths from cervical and uterine body cancer^16^ (ICD-9 182; ICD-10 C54). Corrections were applied proportionally to the registered deaths according to calendar year, place of residence and age group.

For each locality and calendar year in the period 1980–2020, age-standardized mortality rates were calculated, considering data without and with correction, using as standard the world population proposed by Segi^17^ (1960). In addition to standardized rates, age-specific rates were calculated for ages 25–39, 40–64 and 65 years or older.

To estimate the general and specific mortality trend, a linear regression model was applied, according to the methodology used in a previously published study^18^ . Since these are time series with trends that vary over time in a non-regular manner, a linear model for the overall trend for the entire period would not be adequate. In order to consider the existence of structural breaks, the time variable was introduced into the model by means of piecewise linear splines that allowed the identification of inflection moments in the series. With this, models with different break points around the points identified with the splines were tested. The models were compared using Akaike’s criterion (AIC)^19^ to define the points that offered the best fit to the model.

The residuals-based evaluation of the models was performed to verify if the usual assumptions were met, and to check for residual autocorrelation by autocorrelation functions (FAC) and partial autocorrelation (FACP). Models that showed significant autocorrelation or with an absolute value greater than 0.5 were re-estimated using generalized least squares with first-order autoregressive model AR (1), allowing the modeling of autocorrelation and correction of the variance from coefficient estimators. The function “gls” with restricted maximum likelihood estimation (REML) from the package “nlme”^20^ was used.

The coefficient of the term for each segment expresses the logarithm of the trend in that interval. Thus, the annual percent change (APC) of mortality rates was calculated by the formula, with respective 95% confidence intervals (95%CI) and p values. For the interpretation of trends, statistical non-significance (p-value above 0.05) was used as a criterion to characterize an APC as stable. The statistically significant PCA, when positive, indicated an increasing trend, and when negative, a decreasing trend.

The coverage of screening by cytopathological exam of the uterine cervix (Pap Smear) in women aged 25–39 years, 40–64 and 65 years or older in 2009–2020 was evaluated by the ratio between the total number of exams with codes 0203010019 (cervical-vaginal/microflora), and 0203010086 recorded in the SUS^21^ Outpatient Information System ( *Sistema de Informações Ambulatoriais* , SIA) and 1/3 of the female population^13^ excluding the percentage of beneficiaries of health insurance plans, in each age group and locality, obtained from the Brazilian National Agency for Supplementary Health^22^ . Dividing the population into 1/3 is justified by the recommendation that an exam be performed every three years^23^ .

All analyses were performed in the R Software, version 4.1.0.

## RESULTS

In the Southeast region, between 1980 and 2020, there were 63,889 deaths from malignant neoplasm of the cervix (without correction), 665,231 deaths with an ill-defined or unknown underlying cause, and 41,006 deaths from malignant neoplasm of uterus, part unspecified. In Brazil, the respective numbers of deaths were 165,087, 2,178,355 and 83,748.

The correction with redistribution of deaths from an ill-defined or unknown underlying cause led to an increase of 15.38% in the rates for Brazil in 1980, ranging from 7.69% in the Southeast to 35.56% in the Northeast. The increase with this correction was smaller in 2020 (for Brazil 2.17%; 1.96% in the Midwest to 4.21% in the North). Adding to this correction the redistribution of deaths from cervical cancer in the unspecified portion, the rates in 1980 increased by 68.33% for Brazil (ranging from 36.36% in the Midwest to 98.18% in the South). By 2020, smaller increases occurred (for Brazil 19.15%; ranging from 12.12% in the North to 28.57% in the Southeast) (data not shown).

Comparing age-adjusted (with proportional redistribution of deaths from ill-defined causes and redistribution of deaths classified as uterine, SOE) and age-standardized mortality rates, the highest ones were in the North region and the lowest in the Southeast, with a ratio between these in 2020 of 2.47 ( Table 1 ).

**Table 1:** Cervical cancer mortalitya rates without and with correction. Brazil, regions and states in the Southeast region, 1980 and 2020.

Total	Cervical cancer death rates						Rate ratio^c^

	without correction		with correction^b^				

			Correction 1		Correction 2		
			
	1980	2020	1980	2020	1980	2020	2020
Brazil	5.24	4.56	6	4.71	10.13	5.64	1.24
North	8.2	9.49	9.79	9.9	15.08	11.09	2.47
Northeast	4.54	5.55	6.09	5.74	8.92	6.68	1.49
Southeast	5.24	3.38	5.56	3.5	10.04	4.46	1
Minas Gerais	4.43	2.89	5.14	3.02	10.25	3.94	0.87
Espírito Santo	3.21	5.62	3.86	5.66	5.89	6.63	1.47
Rio de Janeiro	5.36	4.34	5.5	4.55	10.28	6.04	1.33
São Paulo	5.71	3.05	5.91	3.14	10.09	3.93	0.87
South	5.08	4.25	5.54	4.34	10.95	5.19	1.16
Midwest	8.03	5.14	8.8	5.23	12.04	5.93	1.31

^a^Age-standardized rates per 100,000 population for the world standard population^17^. International Classification of Diseases 9th (ICD-9) and 10th revision (ICD-10) codes: Cervix (ICD-9 780-799; ICD-10 C53).

^b^ Corrected rates: Correction 1; with proportional redistribution of deaths from ill-defined causes (symptoms, signs, and abnormal findings of clinical and laboratory tests, not elsewhere classified: ICD-9 780-799; ICD-10 R00-R99); Correction 2: with proportional redistribution of deaths from ill-defined causes + redistribution of deaths classified as uterus, SOE (uterus, SOE: ICD-9 179; ICD-10 C55).

^c^Ratio between the mortality rates obtained by Method B in 2020 and the rate of the Southeast region in 2020.

In Brazil, a decrease in the magnitude of mortality rates was observed between 1980 and 2020 ( Table 2 ), a pattern similar to that observed in the Southeast region until 2014. According to the mortality trend analysis in the country, statistically significant declines from 1992–1998 (APC = -0.993; 95%CI -1.767– -0.212) were maintained until 2005–2014 (APC = -2.604; 95%CI -3.108 – -2.097), while in 2014–2020 the trend stabilized (APC = -0.205; 95%CI -1.131–0.730). Rio de Janeiro showed a similar pattern to Brazil, while Minas Gerais expressed a declining trend throughout the 1980–2020 period, despite stability in 1987–1993 and a more modest drop in 2011–2020 (APC = -1.516; 95%CI -2.290–0.735). In São Paulo, starting in 2014, a slight increase in the trend of mortality rates was observed (APC = 1.237; 95%CI 0.046–2.443) ( Table 2 ).

**Table 2:** Annual percentage change (APC) of mortality ratesa correctedb by cervical cancer. Brazil, Southeastern region and states of the Southeastern region, 1980 to 2020.

Geographic Area	Period	Mortality Rates (beginning-end of period)	APC^c^ (%)	95%CI	p
Brazil	1980 **–** 1983	10.13–9.73	-1.872	-3.949-0.249	0.083
	1983 **–** 1992	9.73–9.09	-0.403	-0.963-0.160	0.16
	**1992–1998**	**9.09–8.80**	**-0.993**	**-1.767 – -0.212**	**0.013**
	**1998–2005**	**8.80–7.80**	**-2.025**	**-2.677 – -1.368**	**< 0.001**
	**2005–2014**	**7.80–5.88**	**-2.604**	**-3.108 – -2.097**	**< 0.001**
	2014–2020	5.88–5.64	-0.205	-1.131-0.730	0.666
Southeast Region	**1980–1987**	**10.04–8.40**	**-1.974**	**-2.778 – -1.163**	**< 0.001**
	1987–1995	8.40–8.91	0.31	-0.226-0.849	0.257
	**1995–2014**	**8.91–4.42**	**-3.494**	**-3.698 – -3.290**	**< 0.001**
	2014–2020	4.42–4.46	0.032	-0.848-0.920	0.944
Minas Gerais	**1980–1987**	**10.25–8.17**	**-2.411**	**-3.633 – -1.174**	**< 0.001**
	1987–1993	8.17–8.57	-0.549	-1.637-0.551	0.327
	**1993–2011**	**8.57–4.29**	**-3.256**	**-3.575 – -2.937**	**< 0.001**
	**2011–2020**	**4.29–3.94**	**-1.516**	**-2.290-0.735**	**< 0.001**
Espírito Santo	**1980–1986**	**5.89–10.25**	**5.625**	**2.214–9.149**	**0.001**
	1986–1995	10.25–14.91	1.348	-0.234-2.956	0.095
	**1995–2016**	**14.91–5.15**	**-3.162**	**-3.770 – -2.550**	**< 0.001**
	2016–2020	5.15–6.63	2.434	-2.374-7.478	0.327
Rio de Janeiro	**1980–1987**	**10.28–7.62**	**-2.338**	**-3.792 – -0.861**	**0.002**
	**1987–1994**	**7.62–9.65**	**1.974**	**0.640–3.325**	**0.004**
	**1994–2000**	**9.65–8.37**	**-1.591**	**-3.053 – -0.106**	**0.036**
	**2000–2008**	**8.37–7.07**	**-2.887**	**-3.983 – -1.778**	**< 0.001**
	**2008–2014**	**7.07–5.95**	**-2.125**	**-3.630 – -0.596**	**0.007**
	2014–2020	5.95–6.04	-0.458	-2.286-1.404	0.628
São Paulo	**1980–1999**	**10.09–7.89**	**-1.175**	**-1.446 – -0.904**	**< 0.001**
	**1999–2014**	**7.89–3.76**	**-4.699**	**-5.020 – -4.378**	**< 0.001**
	**2014–2020**	**3.76–3.93**	**1.237**	**0.046–2.443**	**0.042**

95%CI: confidence interval of 95%.

^a^Age-standardized rates per 100,000 population for the world standard population^17^.

^b^ Corrected rates for ill-defined causes (ICD-9 780-799; ICD-10 R00-R99) and uterus, SOE (ICD-9 179; ICD-10 C55).

^c^APC (annual percent change) statistically different from zero in bold.

**Figure 1:**
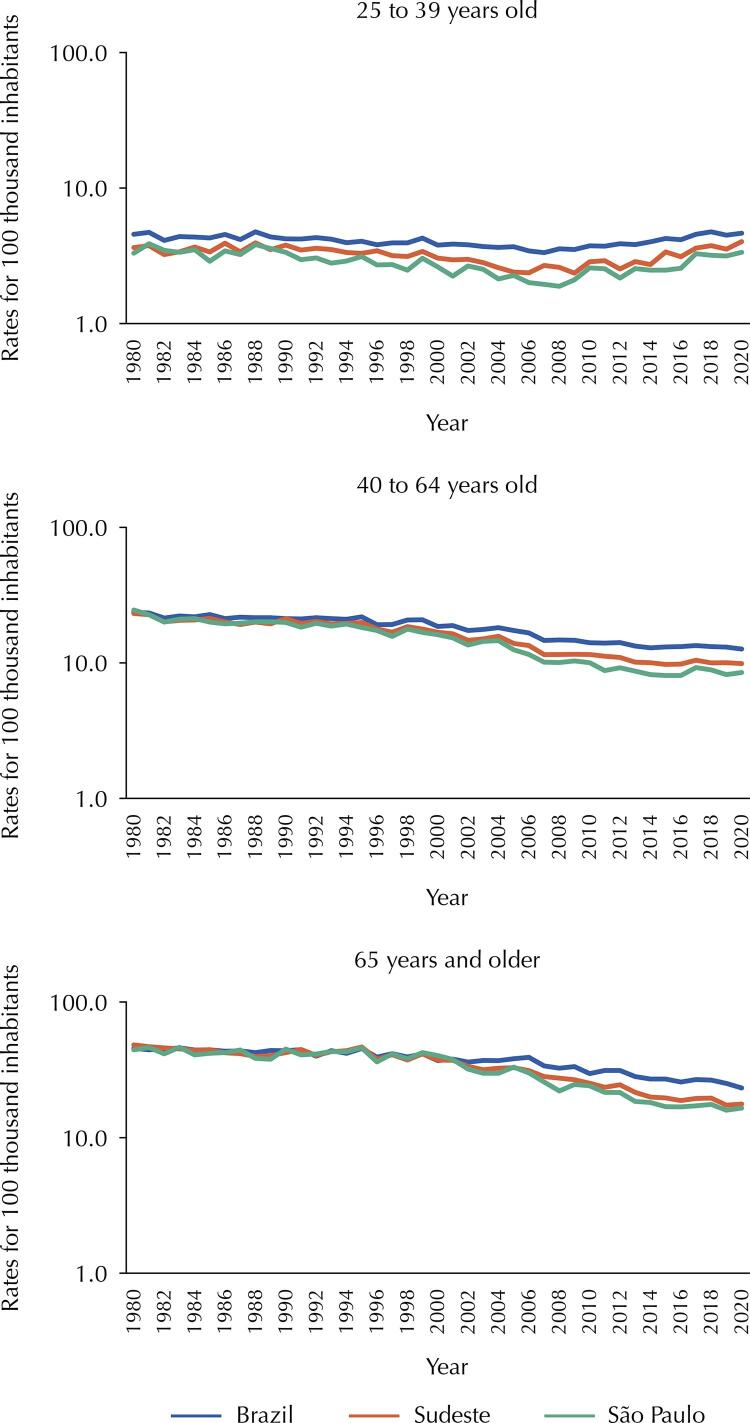
Time trend of mortalitya rates correctedb by cervical cancer specific by age. Brazil, Southeastern region and State of São Paulo, 1980 to 2020. ^a^Rates per 100,000 inhabitants; ^b^Rates corrected for ill-defined causes (ICD-9 780-799; ICD-10 R00-R99) and uterus, SOE (ICD-9 179; ICD-10 C55) presented graphically on a logarithmic scale (log 10).

The temporal distribution of age-specific adjusted mortality rates shows that, in general, the Southeast region and the state of São Paulo had lower rates compared to the country. This divergence is particularly noticeable from the year 2000 onwards. Even though the lowest mortality rates were observed in the 25–39 age group, the increase in the risk of death in this age group is noteworthy in all spatial cuts examined ( Figure 1 ). The trend analysis showed that this increase was evident in the Southeast region in 2013–2020 (APC = 5.072; 95%CI 3.971–6.185) ( Table 3 ). Of less intensity and earlier, the increase in Brazil was detected in 2007–2020 (APC = 2.520; 95%CI 2.092–2.950) and in São Paulo in 2008–2020 (APC = 4.173; 95%CI 3.231–5.123). Minas Gerais stands out for the sharpest upward trend (2011–2020: APC = 6.739; 95%CI 3.611–9.962) ( Table 3 ).

**Table 3:** Annual percentage change (APC)a of mortality rates correctedb by cervical cancer specific by age. Brazil, Southeastern region and states of the Southeastern region, 1980 to 2020.

Total	25 to 39 years old				40 to 64 years old				65 years or older			
		
	Period	APC (%)	95%CI^c^	p	Period	APC (%)	95%CI^c^	p	Period	APC (%)	95%CI^c^	p
Brazil	1980–1989	-0.373	-1.023–0.282	0.264	**1980–1999**	**-0.711**	**-0.930 – -0.491**	**< 0.001**	**1980–1995**	**-0.44**	**-0.798 – -0.079**	**0.017**
	**1989–1999**	**-0.886**	**-1.384 – -0.385**	**0.001**	**1999–2011**	**-3.075**	**-3.396 – -2.753**	**< 0.001**	**1995–2008**	**-1.692**	**-2.050 – -1.333**	**< 0.001**
	**1999–2007**	**-1.803**	**-2.392 – -1.210**	**< 0.001**	**2011–2020**	**-0.773**	**-1.346 – -0.197**	**0.009**	**2008–2020**	**-2.925**	**-3.391 – -2.456**	**< 0.001**
	**2007–2020**	**2.52**	**2.092–2.950**	**< 0.001**	-	-	-	-	-	-	-	-
Southeast Region	1980–1989	0.55	-0.172–1.276	0.136	**1980–1986**	**-1.949**	**-3.212 – -0.670**	**0.003**	**1980–1986**	**-2.753**	**-4.106 – -1.380**	**< 0.001**
	**1989–1999**	**-1.331**	**-1.872 – -0.787**	**< 0.001**	1986–1995	-0.128	-0.760-0.507	0.691	**1986–1995**	**0.823**	**0.164–1.487**	**0.014**
	**1999–2006**	**-3.822**	**-4.602 – -3.036**	**< 0.001**	**1995–2013**	**-3.607**	**-3.889 – -3.324**	**< 0.001**	**1995–2020**	**-3.723**	**-3.935 – -3.510**	**< 0.001**
	**2006–2013**	**2.056**	**1.169–2.951**	**< 0.001**	2013–2020	-0.366	-1.329-0.606	0.459	-	-	-	-
	**2013–2020**	**5.072**	**3.971–6.185**	**< 0.001**	-	-	-	-	-	-	-	-
Minas Gerais	1980–1996	-1.169	-2.482–0.161	0.085	**1980–1993**	**-1.455**	**-2.206 – -0.697**	**< 0.001**	**1980–1986**	**-3.24**	**-5.599 – -0.822**	**0.009**
	**1996–2005**	**-3.867**	**-6.067 – -1.616**	**0.001**	**1993–2009**	**-3.655**	**-4.165 – -3.142**	**< 0.001**	1986–1995	-0.207	-1.619-1.226	0.776
	2005–2011	-0.406	-4.126–3.458	0.834	**2009–2020**	**-1.663**	**-2.589 – -0.727**	**0.001**	**1995–2000**	**-4.661**	**-7.092 – -2.165**	**< 0.001**
	**2011–2020**	**6.739**	**3.611–9.962**	**< 0.001**	-	-	-	-	2000–2006	-0.705	-2.704-1.334	0.495
	-	-	-	-	-	-	-	-	**2006–2020**	**-4.023**	**-4.857 – -3.181**	**< 0.001**
Espírito Santo	**1980–1987**	**15.411**	**8.505–22.756**	**< 0.001**	**1980–1995**	**2.501**	**1.251–3.766**	**< 0.001**	**1980–1984**	**12.187**	**2.790–22.444**	**0.01**
	1987–2000	-0.6	-3.046–1.909	0.636	**1995–2013**	**-3.963**	**-4.844 – -3.073**	**< 0.001**	1984–1995	0.165	-1.926-2.301	0.878
	**2000–2016**	**-2.154**	**-4.154 – -0.113**	**0.039**	2013–2020	0.635	-2.546–3.920	0.699	**1995–2020**	**-2.369**	**-3.198 – -1.533**	**< 0.001**
	2016–2020	11.473	-1.046–25.575	0.074	-	-	-	-	-	-	-	-
Rio de Janeiro	**1980–1991**	**2.366**	**0.742–4.016**	**0.004**	1980–1987	-1.522	-3.367–0.357	0.112	**1980–1987**	**-4.36**	**-6.628 – -2.036**	**< 0.001**
	1991–2000	0.032	-1.777–1.874	0.973	**1987–1993**	**2.045**	**0.118–4.008**	**0.037**	**1987–1995**	**2.152**	**0.626–3.700**	**0.006**
	**2000–2005**	**-5.741**	**-8.641 – -2.749**	**< 0.001**	1993–2000	-0.815	-2.365–0.759	0.308	**1995–2020**	**-3.589**	**-4.025 – -3.151**	**< 0.001**
	**2005–2020**	**3.142**	**1.988–4.309**	**< 0.001**	**2000–2008**	**-2.839**	**-4.088 – -1.575**	**< 0.001**	-	-	-	-
	-	-	-	-	**2008–2018**	**-1.852**	**-2.956 – -0.735**	**0.001**	-	-	-	-
	-	-	-	-	2018–2020	5.787	-2.091–14.298	0.154	-	-	-	-
São Paulo	1980–1988	0.04	-1.463–1.565	0.959	**1980–1985**	**-3.109**	**-4.937 – -1.245**	**0.001**	1980–1989	-1.119	-2.310-0.086	0.069
	**1988–2008**	**-2.595**	**-3.045 – -2.143**	**< 0.001**	1985–1993	-0.543	-1.500–0.422	0.269	1989–1995	0.794	-0.939-2.558	0.371
	**2008–2020**	**4.173**	**3.231–5.123**	**< 0.001**	**1993–2001**	**-2.44**	**-3.352 – -1.521**	**< 0.001**	1995–1999	-1.404	-3.689-0.936	0.237
	-	-	-	-	**2001–2007**	**-5.879**	**-7.102 – -4.641**	**< 0.001**	**1999–2015**	**-5.105**	**-5.623 – -4.585**	**< 0.001**
	-	-	-	-	**2007–2013**	**-4.076**	**-5.362 – -2.772**	**< 0.001**	2015–2020	-1.139	-3.285-1.055	0.306
	-	-	-	-	2013–2020	0.073	-1.231-1.394	0.913	-	-	-	-

95%CI: confidence interval of 95%.

^a^APC (Annual Percent Change) statistically different from zero in bold.

^b^Corrected rates for ill-defined causes (ICD-9 780-799; ICD-10 R00-R99) and uterus, SOE (ICD-9 179; ICD-10 C55).

In the 40–64 age group, marked declines occurred in both Brazil and the Southeast region, as well as in São Paulo between the years 1990 and 2013, after which mortality rates remained decreasing in the country but became stable in the Southeast and São Paulo. In ages 65 years or older, it is worth noting the prominent decline in mortality in São Paulo in the 1999–2015 period (APC = -5.105; 95%CI -5.623 – -4.585), followed by stability in 2015–2020 (APC = -1.139; 95%CI -3.285–1.055) ( Table 3 ).

**Figure 2:**
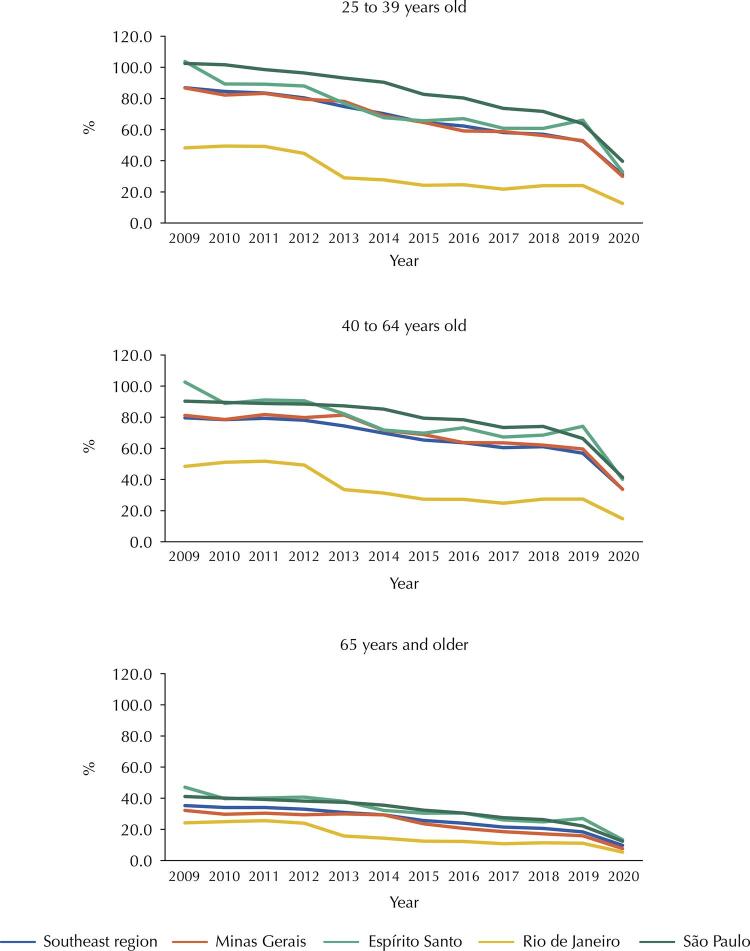
Coveragea (%) by Pap Smear exams in women targeted for screening (25–39 and 40–64 years) and non-targeted (65 years and over) in SUS. Southeastern region and states, 2009 to 2020. ^a^Ratio of cervical cytopathological exams to 1/3 of the female population using the SUS for each age group multiplied by 100. This indicator is considered a proxy for screening test coverage^23^.

Regarding the performance of cervical cytopathological exam in the Southeast in 2009–2020, the highest coverage was observed in São Paulo and the lowest in Rio de Janeiro ( Figure 2 ). In all age groups, coverage remained higher until 2012, however, uneven. In the 25–39 age group, the average coverage for the 2009–2012 period remained above 80% for the Southeastern states, except in Rio de Janeiro (48%). In the 40–64 age group, the evolution of rates was similar to the previous age group. There was a consistent drop in coverage as of 2012 at all ages, worsening in 2020. In women 65 years or older (the age group outside of screening), lower coverage rates were seen with similar time evolution as the younger groups ( Figure 2 ).

## DISCUSSION

The Brazilian scenario is compatible with the inverse correlation between the occurrence of cervical cancer and the level of socioeconomic development^24^ . The highest mortality rates were observed in regions with lower socioeconomic status^25^ and less access to health services, such as the North and Northeast regions^26^ . In four decades there was a downward trend throughout the country, with the exception of the interior of the North region, which in 2017 showed rates three times higher than the Southeast, unveiling the extreme inequality in the risk of becoming ill and dying from this cancer^18^ .

Although the downward curves in mortality may be attributed to the greater health equity resulting from the implementation of the SUS in 1990^27^ and, to some extent, to the opportunistic screening started in 1998, the speed of the decline in mortality was slower than that observed in other Latin American countries such as Chile^28^ .

In the United States, the decline in incidence and mortality has also been observed with the implementation of cytological screening, but many racial and socioeconomic inequalities exist^29^ . In the Latin American and Caribbean region and Asia, the incidence of cervical cancer is relatively high. Favorable trends in incidence have been observed in several countries; however, but preventive actions are inefficient and probably this decrease is related to other factors such as decreased fertility and birth rate, hygiene conditions, or improved socioeconomic status^30^ . In Brazil, Chile, and Colombia, positive outlook for cervical cancer is related to better structured screening programs and relatively higher coverage rates than other Latin American countries^31^ , although the impact of these programs is limited by inequalities in access to diagnostic and treatment services and suboptimal coverage and follow-up rates^32^ .

In this study, the trend of increasing cervical cancer mortality among women aged 25–39 years in Brazil was very pronounced in the Southeastern states, especially Minas Gerais and Sao Paulo. This phenomenon has been seen in other countries recently^33^ . In São Paulo, the increase observed in the 25–39 age group (also observed in Minas Gerais and Rio de Janeiro), stability in the 40–64 age group (also seen in Espírito Santo and Rio de Janeiro), and the only one to show stability in ages 65 years or older are noteworthy. This finding points out that São Paulo is the first Brazilian state to show a reversal trend in mortality from cervical cancer.

The increased incidence and mortality among younger women has been attributed to changes in sexual behavior that increase the risk of persistent HPV infection^33^ . Despite the finding that there it is a cancer in decline in several countries, recent trends of increasing incidence among young women, more marked in high-income countries, have been observed^34 , 35^ . For birth cohorts from 1940 or 1950 onwards an increase in incidence was observed in European countries and Japan, while the incidence remained stable in the United States^3^ . The same has been described in Central Europe, Eastern Europe, and Central Asia^33^ . This increase has raised debate around the need to review and implement more effective screening strategies.

At younger ages, cancer mortality rates are lower than those at older ages, and are therefore more prone to fluctuations due to fewer deaths. In this study, the trend of increasing mortality among young women was verified through positive and statistically significant annual percentage changes. For this reason, we consider it relevant to show this situation that has been reported in other countries. In Brazil, this finding demands attention to the screening coverage indicators in this specific age group, as well as to indicators of access to diagnosis and treatment, since fewer cervical cancer deaths are expected at these age groups. Knowing the prevalence of HPV infection over time and whether the disease has affected these women earlier would bring important contributions to the direction of specific actions at all levels of healthcare.

The self-reported coverage of Pap Smear for all women in the target age group was 78.8% in the country^36^ and 80% in the capital cities^37^ , proportions that may be considered high. Since these are based on self-reported information from women interviewed in population surveys, they may not reflect the actual screening coverage. At the same time, the incidence and mortality rates for cervical cancer remain high compared to other countries^2^ , with great disparity between regions^38^ . In this aspect, it is worth noting that the actual coverage is certainly lower than those reported in population surveys.

Lower coverages are observed from the SUS databases - SISCOLO, SISCAN, and SIA. Recently, a drop was identified in the number of women who performed cytopathological exam for the first time in SISCOLO, reaching 41% between the years 2012 and 2013^39^ . Moreover, the availability of exams for diagnostic confirmation in SUS is deficient, which impairs following-up of screened women^40^ .

In the Southeast, especially in São Paulo, SUS screening coverage tends to be higher compared to other states in the country. In 2009–2020, declines in the percentages of screening test coverage were observed in all states and age groups, indicating that access to the exam in the SUS has been reduced. The lowest level of coverage occurred in 2020, which can be explained by the Covid-19 pandemic.

Vale et al.^41^ found a significant reduction in the proportion of exams performed outside the screening target age group in Campinas, a municipality located in the state of São Paulo, between 2010 and 2016, especially in women under 25 years old. According to the authors, the better alignment of local practices with national guidelines could explain the declines observed in coverage rates in recent years. In addition to a reduction in the excess of exams, Vale et al.^41^ found increases in the proportions of exams performed among women aged 25 and 64 years, a fact not observed in this study. The data presented for the Southeast region showed that the reductions observed since 2009 in the group aged 65 and over were also verified in the age group of 25–64 years. This fact suggests there are other factors related to the decline in screening coverage in the Southeast region, in addition to those pointed out in the Campinas study^41^ .

In addition to the decrease in coverage, there are problems in the follow-up of the abnormal results. A study based on linkage of data from SISCAN, SIA, and Hospital Admissions System ( *Sistema de Internações Hospitalares,* SIH) reviewed the quality of follow-up of screened women in the state of São Paulo and found that for 35.2% of women with abnormal cytology, there were no data found on the diagnosis in the information systems^11^ . It also identified a median time greater than six months between the altered test and diagnosis and almost three months between diagnosis and the beginning of treatment. These prolonged times were associated with worse conditions of care in the regional healthcare units of the state. Reinforcing these findings, another study also conducted in São Paulo concluded that access to colposcopy is limited in the state, impairing diagnosis and consequently treatment^42^ .

Delays in diagnosis lead to diagnosis in more advanced stages. In this sense, screening actions for cervical cancer play an important role, not only in reducing the incidence of the disease, but also in reducing mortality by providing early diagnosis.

A study conducted in Brazil, based on data from hospital-based cancer registries, showed that the diagnosis of cervical cancer occurred late (stages III-IV), in 53.5% of cases in 2012^43^ . In São Paulo, hospital data on invasive cervical tumors^44^ diagnosed in 2017 pointed out that 39.2% of cases in women aged 25–39 years were diagnosed in stages III-IV. In the age groups 40–64 and 65 years or older, the respective proportions were 51.7% and 62.3%. These data suggest a worrisome scenario, considering that the national cervical cancer control program has been implemented nationwide since the late 1990s. Improvements in the early detection of this cancer would have an important effect in reducing mortality in the country.

The discovery of the causal role of HPV^1^ entails the need to reformulate primary and secondary prevention of cervical cancer^6^ . The introduction of HPV testing can optimize and make screening more effective, especially in low-income countries^45^ . Data from large randomized studies have shown that protection against invasive carcinoma with screening based on HPV testing from 30 years of age and at 5-year intervals is 60–70% higher compared to oncotic cytology^7^ .

There are issues that deserve investigation in order to make decisions about strategic and cost-effective measures that should be implemented in cervical cancer control programs. Some of these are the definition of screening intervals, ways to motivate women to adhere, and the organization of services from access to screening to improved infrastructure for diagnosis and treatment^46^ . Even with the availability of the HPV test as a recommended screening test, challenges inherent to the organization of the program will continue to exist. More than the screening test, the use of the most appropriate approach to organize all components, including quality aspects, are determining factors for the success of a screening program^8^ .

Analyses of trends of mortality from cervical cancer are often impaired by inaccuracies when filling in the underlying cause of death, since a portion is registered as malignant neoplasm of uterus, part unspecified, which does not allow knowledge of the true anatomical origin of the tumor (cervix or body of the uterus). To deal with this limitation, a technique was employed to correct the deaths from cervical cancer originally recorded in the SIM, allowing more realistic analyses. It should be highlighted that in 2020 the correction for ill-defined causes was low in all regions (ranged from 1.96% in the Midwest to 4.21% in the North). However, the redistribution by uterus part unspecified led to an increase of 28.57% in the Southeast region, a higher percentage if compared to the other regions.

After significant progress in the accuracy of death information in the 1980–2020 period and decades of decline in mortality from cervical cancer, recent trends of stability in Brazil and increase in the state of São Paulo point to the need for reorganizing the current screening program to achieve improvements in coverage and quality in all its stages - screening, diagnosis, and treatment. Immunization against HPV will bring positive results in the long term, but its implementation does not minimize the role of secondary prevention; rather, it reinforces the immediate need for planning for implementation in the medium term of a more cost-effective and sensitive screening test.

An organized screening program will make it possible to actively reach women in the target age group, and especially women 25–39 years old, in which a sharp increase in mortality from cervical cancer has been observed in the country. These are women in full sexual activity and also clearly integrated into the economically active population and, thus, with greater difficulties in adhering to screening.

Only with a broad approach including high coverage, quality of examinations, and follow-up throughout the cancer care pathway, will greater reductions not only in mortality, but also in incidence be achieved. This will ensure Brazil’s alignment with the global strategy of eliminating cervical cancer as a public health issue^47^ .
