# Supplementary material for: Recent changes in trends of mortality from cervical cancer in Southeastern Brazil
Source: Rev Saude Publica. 2023 Aug 28;57:25. doi: 10.11606/s1518-8787.2023057004709 (PMC10118416; doi:10.11606/s1518-8787.2023057004709)
Supplement: Translation

# Translation: Mudanças recentes nas tendências da mortalidade por câncer de colo do útero no Sudeste do Brasil

**Authors:** Carolina Terra de Moraes Luizaga, Beatriz Cordeiro Jardim, Victor Wünsch, José Eluf, Gulnar Azevedo e Silva

**Affiliations:** I Brasil Fundação Oncocentro de São Paulo. São Paulo, SP, Brasil; II Brasil Universidade do Estado do Rio de Janeiro. Instituto de Medicina Social Hesio Cordeiro. Rio de Janeiro, RJ, Brasil; III Brasil Instituto Nacional de Câncer. Rio de Janeiro, RJ, Brasil; IV Brasil Universidade de São Paulo. Faculdade de Saúde Pública. São Paulo, SP, Brasil; V Brasil Universidade de São Paulo. Faculdade de Medicina. São Paulo, SP, Brasil

**Keywords:** Neoplasias do Colo do Útero, Registros de Mortalidade, Análise Espaço-Temporal, Estudos de Séries Temporais, Brasil

## Abstract

**OBJETIVO:**

Analisar as tendências da mortalidade por câncer de colo de útero nos estados da região Sudeste e compará-las com o Brasil e demais regiões entre 1980 e 2020.

**MÉTODOS:**

Estudo de série temporal com base nos dados do Sistema de Informações de Mortalidade. Os dados de óbito foram corrigidos por redistribuição proporcional das mortes por causas mal definidas e por câncer de útero de porção não especificada. Foram calculadas taxas padronizadas por idade e específicas por faixas etárias alvo de rastreamento (25–39 anos; 40–64 anos) e não alvo (65 anos ou mais). Variações percentuais anuais foram estimadas por modelo de regressão linear com pontos de quebra. A cobertura do exame Papanicolaou no Sistema Único de Saúde (SUS) foi avaliada entre 2009 e 2020 segundo faixa etária e localidade.

**RESULTADOS:**

Foram verificados aumentos das taxas de mortalidade corrigidas tanto em 1980 como em 2020 em todas as regiões, com incrementos mais evidentes no início da série. Houve queda da mortalidade em todo o país entre 1980–2020, entretanto, o estado de São Paulo apresentou discreta tendência de aumento em 2014–2020 (APC=1,237; IC95% 0,046–2,443). Destaca-se o incremento da tendência no grupo de 25–39 anos em todas as localidades de estudo, mostrando-se mais acentuado na região Sudeste em 2013–2020 (APC=5,072; IC95% 3,971–6,185). As taxas de cobertura de rastreamento foram mais elevadas em São Paulo e mais baixas no Rio de Janeiro, com queda consistente a partir de 2012 em todas as idades.

**CONCLUSÕES:**

São Paulo é o primeiro estado brasileiro a apresentar inversão de tendência da mortalidade por câncer de colo do útero. As mudanças nos padrões de mortalidade identificadas neste estudo apontam para a necessidade de reorganização do atual programa de rastreamento, que deve ser aperfeiçoado para garantir alta cobertura, qualidade e seguimento adequado de todas as mulheres com exames alterados.

## INTRODUÇÃO

O câncer de colo do útero é uma doença que tem como causa necessária a infecção persistente por tipos de papilomavírus humano (HPV) de alto risco^1^ e, apesar de potencialmente evitável, ainda é um grave problema de saúde em países de média e baixa renda^2^ . Com grandes variações globais nas taxas de mortalidade, ele se apresenta como o principal tipo de câncer relacionado ao óbito entre mulheres em 36 países^2^ . Nas últimas décadas, reduções expressivas na mortalidade e incidência ocorreram em países que implementaram programas de rastreamento baseados na citologia vaginal pelo Papanicolaou^3^ , com melhores resultados encontrados naqueles com rastreamento organizado^4^ .

Adversamente, alguns países que tiveram importante queda na morbimortalidade decorrente do rastreamento organizado com base no Papanicolaou começaram a mostrar quedas em menor intensidade na mortalidade pela doença, estabilidade ou mesmo aumento^5 - 6^ , levando à necessidade de investimentos em estratégias mais eficientes para organização dos programas de rastreamento^7 - 8^ .

As primeiras iniciativas de detecção precoce do câncer de colo do útero no Brasil foram isoladas, com populações restritas e ocorreram no final da década de 1980. Somente a partir de 1998, com o desenvolvimento de um programa de controle desse câncer pelo Ministério da Saúde (MS), as práticas de rastreamento foram estruturadas em caráter oportunístico em todo o território nacional. As diretrizes atuais recomendam o Papanicolaou para mulheres de 25–64 anos de idade^9^ . Para monitoramento dos exames de rastreamento e de confirmação diagnóstica no Sistema Único de Saúde (SUS), o MS implantou sistemas de informações denominados SISCOLO (Sistema de Informação do Câncer do Colo do Útero) e SISCAN (Sistema de Informação do Câncer).

No estado de São Paulo, dados disponíveis na Secretaria Estadual de Saúde indicam declínio na cobertura dos exames de Papanicolaou realizados no SUS a partir de 2010^10^ e problemas no seguimento de mulheres rastreadas^11^ , o que pode impactar a tendência de queda da mortalidade a médio e longo prazos. Este estudo tem o objetivo de analisar as tendências da mortalidade do câncer de colo do útero nos estados da região Sudeste e compará-las com o Brasil e demais regiões entre 1980 e 2020.

## MÉTODOS

Estudo de série temporal utilizando dados dos óbitos entre mulheres registrados no Sistema de Informação sobre Mortalidade (SIM) no período 1980–2020. Os dados foram obtidos na página do DATASUS^12^ , sendo os dados de 2020 ainda preliminares. Para 1980-1995 foi utilizada a nona edição da Classificação Estatística Internacional de Doenças, Lesões e Causas de Óbito (CID-9) e entre 1996–2020 a décima edição (CID-10).

As populações para cada localidade foram obtidas a partir de tabulações no DATASUS^13^ de acordo com: 1) 1980–2012 dados dos Censos (1980, 1991, 2000 e 2010), Contagem (1996) e Projeções intercensitárias (1981–2012); e 2) 2013–2020 dados do Estudo de Estimativas Populacionais.

Os dados de mortalidade e populações foram agregados em intervalos de 5 em 5 anos a partir dos 15 até 79 anos de idade (0–14, 15–19, 20–24, 25–29, 30–34, 35–39, 40–44, 45–49, 50–54, 55–59, 60–64, 65–69, 70–74, 75–79 e 80 anos ou mais) segundo Unidade da Federação para o Sudeste, Brasil e regiões brasileiras de residência. Os óbitos sem informação de idade foram proporcionalmente distribuídos entre os sete grupos etários de acordo com a causa básica de morte, local de residência e ano de óbito.

Foi realizada correção da informação sobre a causa básica de óbito seguindo metodologia proposta por Mathers et al.^14^ (2003) e adaptação feita por Girianelli et al.^15^ (2014), que consiste em redistribuir proporcionalmente 50% dos óbitos com causa básica mal definida (CID-9 780-799; CID-10 R00-R99). Esta correção está identificada neste estudo como Correção 1.

Para os óbitos registrados como câncer de colo do útero (CID-9 180; CID-10 C53), realizou-se correção adicional (Correção 2), com redistribuição dos óbitos classificados como neoplasia maligna do útero de porção não especificada (CID-9 179; CID-10 C55), mantendo a proporção registrada como óbitos por câncer de colo e de corpo uterino^16^ (CID-9 182; CID-10 C54). As correções foram aplicadas de maneira proporcional aos óbitos registrados segundo ano-calendário, local de residência e faixa etária.

Para cada localidade e ano-calendário em 1980-2020 foram calculadas as taxas de mortalidade padronizadas por idade, considerando os dados sem correção e com correção, tendo como padrão a população mundial proposta por Segi^17^ (1960). Além das taxas padronizadas, foram calculadas as taxas específicas por faixas etárias de 25-39 anos, 40–64 e 65 anos ou mais.

Para estimar a tendência da mortalidade geral e específica aplicou-se um modelo de regressão linear, conforme metodologia utilizada em estudo previamente publicado^18^ . Por se tratar de séries temporais com tendências que variam no tempo de forma não regular, um modelo linear para tendência global para todo o período não seria adequado. Para considerar a existência de quebras estruturais, a variável tempo foi introduzida ao modelo por meio de *spline* linear por partes que permitiram identificar os momentos de inflexão na série. Com isso, foram testados modelos com diferentes pontos de quebra no entorno dos pontos identificados com as *splines* . Os modelos foram comparados pelo critério de Akaike (AIC)^19^ para definir os pontos que ofereceram melhor ajuste ao modelo.

A avaliação dos modelos via resíduos foi realizada para verificar se os pressupostos usuais foram atendidos e para verificar a existência de autocorrelação residual por funções de autocorrelação (FAC) e autocorrelação parcial (FACP). Os modelos que apresentaram autocorrelação significativa ou com valor absoluto maior que 0,5 foram estimados novamente usando mínimos quadrados generalizados com modelo autorregressivo de primeira ordem AR (1), permitindo modelar a autocorrelação e corrigir a variância dos estimadores dos coeficientes. Foi utilizada a função “gls” com estimação por máxima verossimilhança restrita (REML) do pacote “nlme”^20^ .

O coeficiente do termo referente a cada segmento expressa o logaritmo da tendência naquele intervalo. Assim, foram calculadas as variações percentuais anuais ( *annual percent change* – APC) das taxas de mortalidade pela fórmula, com respectivos intervalos de confiança de 95% (IC95%) e valores de p. Para a interpretação das tendências, a não significância estatística (p-valor acima de 0,05) foi utilizada como critério para caracterizar uma APC como estável. A APC estatisticamente significante, quando positiva, indicou tendência crescente e, quando negativa, decrescente.

A cobertura de rastreamento pelo exame citopatológico do colo uterino (Papanicolaou) em mulheres de 25-39 anos, 40-64 e 65 anos ou mais em 2009-2020 foi avaliada pela razão entre o número total de exames com códigos 0203010019 (cérvico-vaginal/microflora) e 0203010086 registrados no Sistema de Informações Ambulatoriais do SUS^21^ (SIA) e 1/3 da população feminina^13^ excluindo-se o percentual de beneficiárias de planos de saúde, em cada grupo etário e localidade, obtido da Agência Nacional de Saúde Suplementar^22^ . A divisão da população a 1/3 justifica-se pela recomendação de que um exame seja realizado a cada três anos^23^ .

Todas as análises foram realizadas no Programa R, versão 4.1.0.

## RESULTADOS

Na região Sudeste, entre 1980 e 2020 ocorreram 63.889 mortes por neoplasia maligna do colo do útero (sem correção), 665.231 mortes com causa básica mal definida ou desconhecida e 41.006 mortes por neoplasia maligna do útero, porção não especificada. No Brasil, os respectivos números de mortes foram 165.087, 2.178.355 e 83.748.

Pela correção com redistribuição dos óbitos por causa básica mal definida ou desconhecida, as taxas para o Brasil em 1980 aumentaram em 15,38%, variando de 7,69% na região Sudeste a 35,56% no Nordeste. O aumento com essa correção foi menor em 2020 (para o Brasil 2,17%; 1,96% no Centro-Oeste a 4,21% no Norte). Acrescentando-se a esta correção a redistribuição dos óbitos por câncer do útero porção não especificada, as taxas em 1980 aumentaram em 68,33% para o Brasil (variando de 36,36% no Centro-Oeste a 98,18% no Sul). Em 2020, aumentos menores ocorreram (para o Brasil 19,15%; variação de 12,12% na região Norte a 28,57% no Sudeste) (dados não apresentados).

Comparando-se taxas de mortalidade corrigidas (com redistribuição proporcional dos óbitos por causas mal definidas e redistribuição dos óbitos classificados como útero, SOE) e padronizadas por idade, observam-se que as mais altas foram as da região Norte e as mais baixas no Sudeste, com razão entre estas em 2020 de 2,47 ( Tabela 1 ).

**Tabela 1:** Taxas de mortalidade por câncer de colo do úteroa sem e com correção. Brasil, regiões e estados da região Sudeste, 1980 e 2020.

Área	Taxas de mortalidade por câncer de colo do útero						Razão de taxas^c^

	Sem correção		Com correção^b^				

			Correção 1		Correção 2		
			
	1980	2020	1980	2020	1980	2020	2020
Brasil	5,24	4,56	6	4,71	10,13	5,64	1,24
Norte	8,2	9,49	9,79	9,9	15,08	11,09	2,47
Nordeste	4,54	5,55	6,09	5,74	8,92	6,68	1,49
Sudeste	5,24	3,38	5,56	3,5	10,04	4,46	1
Minas Gerais	4,43	2,89	5,14	3,02	10,25	3,94	0,87
Espírito Santo	3,21	5,62	3,86	5,66	5,89	6,63	1,47
Rio de Janeiro	5,36	4,34	5,5	4,55	10,28	6,04	1,33
São Paulo	5,71	3,05	5,91	3,14	10,09	3,93	0,87
Sul	5,08	4,25	5,54	4,34	10,95	5,19	1,16
Centro-Oeste	8,03	5,14	8,8	5,23	12,04	5,93	1,31

^a^Taxas por 100 mil habitantes padronizadas por idade pela população padrão mundial^17^. Códigos da Classificação Internacional de Doenças 9ª (CID-9) e 10ª revisão (CID-10): Colo do útero (CID-9 780-799; CID-10 C53).

^b^Taxas corrigidas: Correção 1; com redistribuição proporcional dos óbitos por causas mal definidas (sintomas, sinais e achados anormais de exames clínicos e de laboratório, não classificados em outra parte: CID-9 780-799; CID-10 R00-R99); Correção 2: com redistribuição proporcional dos óbitos por causas mal definidas + redistribuição dos óbitos classificados como útero, SOE (útero, SOE: CID-9 179; CID-10 C55).

^c^Razão entre as taxas de mortalidade obtidas pelo Método B em 2020 e a taxa da região Sudeste em 2020.

No Brasil, verificou-se queda na magnitude das taxas de mortalidade entre 1980 e 2020 ( Tabela 2 ), padrão semelhante ao observado na região Sudeste até 2014. Pela análise de tendência da mortalidade, no país, declínios estatisticamente significativos a partir de 1992–1998 (APC = -0,993; IC95% -1,767– -0,212) mantiveram-se até 2005-2014 (APC = -2,604; IC95% -3,108 – -2,097), quando em 2014-2020 a tendência se estabilizou (APC = -0,205; IC95% -1,131–0,730). Rio de Janeiro apresentou padrão semelhante ao brasileiro, enquanto Minas Gerais expressou tendência declinante em todo o período 1980-2020, apesar da estabilidade em 1987-1993 e queda mais modesta em 2011-2020 (APC = -1,516; IC95% -2,290–0,735). Em São Paulo, a partir de 2014, observou-se discreto aumento na tendência das taxas de mortalidade (APC = 1,237; IC95% 0,046–2,443) ( Tabela 2 ).

**Tabela 2:** Variação percentual anual (APC) das taxas de mortalidadea corrigidasb por câncer de colo do útero. Brasil, região Sudeste e estados da região Sudeste, 1980 a 2020.

Área geográfica	Período	Taxas de mortalidade (início–fim do período)	APC^c^(%)	IC95%	p
Brasil	1980 **–** 1983	10,13–9,73	-1,872	-3,949–0,249	0,083
	1983 **–** 1992	9,73–9,09	-0,403	-0,963–0,160	0,16
	**1992–1998**	**9,09–8,80**	**-0,993**	**-1,767– -0,212**	**0,013**
	**1998–2005**	**8,80–7,80**	**-2,025**	**-2,677– -1,368**	**< 0,001**
	**2005–2014**	**7,80–5,88**	**-2,604**	**-3,108– -2,097**	**< 0,001**
	2014–2020	5,88–5,64	-0,205	-1,131–0,730	0,666
Região Sudeste	**1980–1987**	**10,04–8,40**	**-1,974**	**-2,778– -1,163**	**< 0,001**
	1987–1995	8,40–8,91	0,31	-0,226–0,849	0,257
	**1995–2014**	**8,91–4,42**	**-3,494**	**-3,698– -3,290**	**< 0,001**
	2014–2020	4,42–4,46	0,032	-0,848–0,920	0,944
Minas Gerais	**1980–1987**	**10,25–8,17**	**-2,411**	**-3,633– -1,174**	**< 0,001**
	1987–1993	8,17–8,57	-0,549	-1,637–0,551	0,327
	**1993–2011**	**8,57–4,29**	**-3,256**	**-3,575– -2,937**	**< 0,001**
	**2011–2020**	**4,29–3,94**	**-1,516**	**-2,290–0,735**	**< 0,001**
Espírito Santo	**1980–1986**	**5,89–10,25**	**5,625**	**2,214–9,149**	**0,001**
	1986–1995	10,25–14,91	1,348	-0,234–2,956	0,095
	**1995–2016**	**14,91–5,15**	**-3,162**	**-3,770– -2,550**	**< 0,001**
	2016–2020	5,15–6,63	2,434	-2,374–7,478	0,327
Rio de Janeiro	**1980–1987**	**10,28–7,62**	**-2,338**	**-3,792– -0,861**	**0,002**
	**1987–1994**	**7,62–9,65**	**1,974**	**0,640–3,325**	**0,004**
	**1994–2000**	**9,65–8,37**	**-1,591**	**-3,053– -0,106**	**0,036**
	**2000–2008**	**8,37–7,07**	**-2,887**	**-3,983– -1,778**	**< 0,001**
	**2008–2014**	**7,07–5,95**	**-2,125**	**-3,630– -0,596**	**0,007**
	2014–2020	5,95–6,04	-0,458	-2,286–1,404	0,628
São Paulo	**1980–1999**	**10,09–7,89**	**-1,175**	**-1,446– -0,904**	**< 0,001**
	**1999–2014**	**7,89–3,76**	**-4,699**	**-5,020– -4,378**	**< 0,001**
	**2014–2020**	**3,76–3,93**	**1,237**	**0,046–2,443**	**0,042**

IC95%: intervalo de confiança de 95%.

^a^Taxas por 100 mil habitantes padronizadas por idade pela população padrão mundial^17^.

^b^Taxas corrigidas para causas mal definidas (CID-9 780-799; CID-10 R00-R99) e útero, SOE (CID-9 179; CID-10 C55).

^c^APC ( *annual percent change* ) estatisticamente diferente de zero em negrito.

A distribuição temporal das taxas de mortalidade corrigidas específicas por faixas etárias mostra que, de forma geral, a região Sudeste e o estado de São Paulo apresentaram taxas menores em comparação com o país. Esse afastamento é particularmente perceptível a partir dos anos 2000. Ainda que as menores taxas de mortalidade tenham sido observadas no grupo de 25–39 anos, destaca-se o aumento do risco de morte nessa faixa etária em todos os recortes espaciais examinados ( Figura 1 ). Pela análise de tendência esse aumento foi evidente na região Sudeste em 2013–2020 (APC = 5,072; IC95% 3,971–6,185) ( Tabela 3 ). De menor intensidade e mais precoce, o aumento no Brasil foi detectado em 2007–2020 (APC = 2,520; IC95% 2,092–2,950) e em São Paulo no período 2008–2020 (APC = 4,173; IC95% 3,231–5,123). Minas Gerais destaca-se pela tendência de aumento mais acentuada (2011–2020: APC = 6,739; IC95% 3,611–9,962) ( Tabela 3 ).

**Figura 1:** Tendência temporal das taxas de mortalidadea corrigidasb por câncer de colo do útero específicas por idade. Brasil, região Sudeste e estado de São Paulo, 1980 a 2020.

^a^Taxas por 100 mil habitantes;^b^Taxas corrigidas para causas mal definidas (CID-9 780-799; CID-10 R00-R99) e útero, SOE (CID-9 179; CID-10 C55) apresentadas graficamente em escala logarítmica (log 10).

**Tabela 3:** Variação percentual anual (APC)a das taxas de mortalidade corrigidasb por câncer de colo do útero específicas por idade. Brasil, região Sudeste e estados da região Sudeste, 1980 a 2020.

Área	25 a 39 anos				40 a 64 anos				65 anos ou mais			
		
	Período	APC (%)	IC95%^c^	p	Período	APC (%)	IC95%^c^	p	Período	APC (%)	IC95%^c^	p
Brasil	1980–1989	-0,373	-1,023–0,282	0,264	**1980–1999**	**-0,711**	**-0,930– -0,491**	**< 0,001**	**1980–1995**	**-0,44**	**-0,798– -0,079**	**0,017**
	**1989–1999**	**-0,886**	**-1,384– -0,385**	**0,001**	**1999–2011**	**-3,075**	**-3,396– -2,753**	**< 0,001**	**1995–2008**	**-1,692**	**-2,050– -1,333**	**< 0,001**
	**1999–2007**	**-1,803**	**-2,392– -1,210**	**< 0,001**	**2011–2020**	**-0,773**	**-1,346– -0,197**	**0,009**	**2008–2020**	**-2,925**	**-3,391– -2,456**	**< 0,001**
	**2007–2020**	**2,52**	**2,092–2,950**	**< 0,001**	-	-	-	-	-	-	-	-
Região Sudeste	1980–1989	0,55	-0,172–1,276	0,136	**1980–1986**	**-1,949**	**-3,212– -0,670**	**0,003**	**1980–1986**	**-2,753**	**-4,106– -1,380**	**< 0,001**
	**1989–1999**	**-1,331**	**-1,872– -0,787**	**< 0,001**	1986–1995	-0,128	-0,760–0,507	0,691	**1986–1995**	**0,823**	**0,164–1,487**	**0,014**
	**1999–2006**	**-3,822**	**-4,602– -3,036**	**< 0,001**	**1995–2013**	**-3,607**	**-3,889– -3,324**	**< 0,001**	**1995–2020**	**-3,723**	**-3,935– -3,510**	**< 0,001**
	**2006–2013**	**2,056**	**1,169–2,951**	**< 0,001**	2013–2020	-0,366	-1,329–0,606	0,459	-	-	-	-
	**2013–2020**	**5,072**	**3,971–6,185**	**< 0,001**	-	-	-	-	-	-	-	-
Minas Gerais	1980–1996	-1,169	-2,482–0,161	0,085	**1980–1993**	**-1,455**	**-2,206– -0,697**	**< 0,001**	**1980–1986**	**-3,24**	**-5,599– -0,822**	**0,009**
	**1996–2005**	**-3,867**	**-6,067– -1,616**	**0,001**	**1993–2009**	**-3,655**	**-4,165– -3,142**	**< 0,001**	1986–1995	-0,207	-1,619–1,226	0,776
	2005–2011	-0,406	-4,126–3,458	0,834	**2009–2020**	**-1,663**	**-2,589– -0,727**	**0,001**	**1995–2000**	**-4,661**	**-7,092– -2,165**	**< 0,001**
	**2011–2020**	**6,739**	**3,611–9,962**	**< 0,001**	-	-	-	-	2000–2006	-0,705	-2,704–1,334	0,495
	-	-	-	-	-	-	-	-	**2006–2020**	**-4,023**	**-4,857– -3,181**	**< 0,001**
Espírito Santo	**1980–1987**	**15,411**	**8,505–22,756**	**< 0,001**	**1980–1995**	**2,501**	**1,251–3,766**	**< 0,001**	**1980–1984**	**12,187**	**2,790–22,444**	**0,01**
	1987–2000	-0,6	-3,046–1,909	0,636	**1995–2013**	**-3,963**	**-4,844– -3,073**	**< 0,001**	1984–1995	0,165	-1,926–2,301	0,878
	**2000–2016**	**-2,154**	**-4,154– -0,113**	**0,039**	2013–2020	0,635	-2,546–3,920	0,699	**1995–2020**	**-2,369**	**-3,198– -1,533**	**< 0,001**
	2016–2020	11,473	-1,046–25,575	0,074	-	-	-	-	-	-	-	-
Rio de Janeiro	**1980–1991**	**2,366**	**0,742–4,016**	**0,004**	1980–1987	-1,522	-3,367–0,357	0,112	**1980–1987**	**-4,36**	**-6,628– -2,036**	**< 0,001**
	1991–2000	0,032	-1,777–1,874	0,973	**1987–1993**	**2,045**	**0,118–4,008**	**0,037**	**1987–1995**	**2,152**	**0,626–3,700**	**0,006**
	**2000–2005**	**-5,741**	**-8,641– -2,749**	**< 0,001**	1993–2000	-0,815	-2,365–0,759	0,308	**1995–2020**	**-3,589**	**-4,025– -3,151**	**< 0,001**
	**2005–2020**	**3,142**	**1,988–4,309**	**< 0,001**	**2000–2008**	**-2,839**	**-4,088– -1,575**	**< 0,001**	-	-	-	-
	-	-	-	-	**2008–2018**	**-1,852**	**-2,956– -0,735**	**0,001**	-	-	-	-
	-	-	-	-	2018–2020	5,787	-2,091–14,298	0,154	-	-	-	-
São Paulo	1980–1988	0,04	-1,463–1,565	0,959	**1980–1985**	**-3,109**	**-4,937– -1,245**	**0,001**	1980–1989	-1,119	-2,310–0,086	0,069
	**1988–2008**	**-2,595**	**-3,045– -2,143**	**< 0,001**	1985–1993	-0,543	-1,500–0,422	0,269	1989–1995	0,794	-0,939–2,558	0,371
	**2008–2020**	**4,173**	**3,231–5,123**	**< 0,001**	**1993–2001**	**-2,44**	**-3,352– -1,521**	**< 0,001**	1995–1999	-1,404	-3,689–0,936	0,237
	-	-	-	-	**2001–2007**	**-5,879**	**-7,102– -4,641**	**< 0,001**	**1999–2015**	**-5,105**	**-5,623– -4,585**	**< 0,001**
	-	-	-	-	**2007–2013**	**-4,076**	**-5,362– -2,772**	**< 0,001**	2015–2020	-1,139	-3,285–1,055	0,306
	-	-	-	-	2013–2020	0,073	-1,231–1,394	0,913	-	-	-	-

IC95%: intervalo de confiança de 95%.

^a^APC ( *annual percent change* ) estatisticamente diferente de zero em negrito.

^b^Taxas corrigidas para causas mal definidas (CID-9 780-799; CID-10 R00-R99) e útero, SOE (CID-9 179; CID-10 C55).

No grupo de 40–64 anos, tanto no Brasil como na região Sudeste e São Paulo, declínios marcantes ocorreram entre os anos 1990 e 2013, a partir de quando as taxas de mortalidade permaneceram em redução no país, mas tornaram-se estáveis no Sudeste e São Paulo. Nas idades de 65 anos ou mais, vale destacar o proeminente declínio na mortalidade em São Paulo no período 1999–2015 (APC = -5,105; IC95% -5,623– -4,585), seguido de estabilidade em 2015–2020 (APC = -1,139; IC95% -3,285–1,055) ( Tabela 3 ).

Em relação à realização do exame citopatológico do colo uterino no Sudeste em 2009–2020, as maiores coberturas foram observadas em São Paulo e as menores no Rio de Janeiro ( Figura 2 ). Em todas as faixas etárias as coberturas mantiveram-se mais elevadas até 2012, entretanto, desiguais. No grupo de 25–39 anos, a cobertura média para o período 2009–2012 manteve-se acima de 80% para os estados do Sudeste, exceto no Rio de Janeiro (48%). Na faixa de 40-64 anos, a evolução das taxas foi semelhante ao grupo etário anterior. Houve queda consistente nas coberturas a partir de 2012 em todas as idades, com agravamento em 2020. Nas mulheres com 65 anos ou mais (grupo etário fora do rastreamento), foram verificadas taxas de cobertura menores com evolução temporal similar aos grupos mais jovens ( Figura 2 ).

**Figura 2:** Coberturaa (%) por exames de Papanicolaou em mulheres alvo de rastreamento (25–39 e 40–64 anos) e não alvo (65 anos ou mais) no SUS. Região Sudeste e estados, 2009 a 2020.

^a^Razão entre exames citopatológicos do colo do útero e 1/3 da população feminina usuária do SUS para cada faixa etária multiplicado por 100. Este indicador é considerado proxy da cobertura dos exames de rastreamento^23^.

## DISCUSSÃO

O cenário brasileiro mostra-se compatível com a correlação inversa entre a ocorrência do câncer de colo do útero e o nível de desenvolvimento socioeconômico^24^ . As maiores taxas de mortalidade foram observadas em regiões de menor nível socioeconômico^25^ e com menor acesso aos serviços de saúde, como Norte e Nordeste^26^ . Em quatro décadas houve tendência de redução em todo o país, com exceção do interior da região Norte que, em 2017, apresentou taxas três vezes mais altas do que o Sudeste, demarcando a extrema desigualdade no risco de adoecimento e morte por esse câncer^18^ .

Embora as curvas descendentes na mortalidade possam ser atribuídas à maior equidade em saúde decorrente da implantação do SUS em 1990^27^ e, em certa medida, ao rastreamento oportunístico iniciado em 1998, a velocidade da queda da mortalidade foi inferior à observada em outros países da América Latina como o Chile^28^ .

Nos Estados Unidos, o declínio da incidência e da mortalidade também foi observado com a implementação do rastreamento citológico, mas muitas desigualdades raciais e socioeconômicas existem^29^ . Na região da América Latina e Caribe e na Ásia, a incidência de câncer de colo do útero é relativamente alta. Tendências favoráveis da incidência foram observadas em vários países, contudo as ações de prevenção são ineficientes e provavelmente essa queda está relacionada a outros fatores como diminuição da fertilidade e natalidade, condições de higiene ou melhora do nível socioeconômico^30^ . No Brasil, Chile e Colômbia, perspectivas positivas do câncer de colo do útero têm relação com programas de rastreamento melhor estruturados e taxas de cobertura relativamente mais altas do que outros países da América Latina^31^ , embora o impacto desses programas seja limitado por desigualdades no acesso aos serviços para diagnóstico e tratamento e taxas de cobertura e seguimento abaixo do ideal^32^ .

Neste estudo, a tendência de aumento da mortalidade pelo câncer de colo do útero entre mulheres de 25-39 anos no Brasil foi bem pronunciada nos estados do Sudeste, em especial em Minas Gerais e São Paulo. Esse fenômeno vem sendo verificado em outros países recentemente^33 - 35^ . Em São Paulo chama atenção o aumento observado na faixa de 25-39 anos (também observado em Minas Gerais e Rio de Janeiro), estabilidade no grupo de 40–64 anos (também visto no Espírito Santo e Rio de Janeiro) e o único a mostrar estabilidade nas idades de 65 anos ou mais. Esse achado indica que São Paulo é o primeiro estado brasileiro a apresentar inversão de tendência de mortalidade pelo câncer de colo do útero.

O aumento da incidência e da mortalidade entre mulheres mais jovens vem sendo atribuído a mudanças no comportamento sexual que aumentam o risco de infecção persistente pelo HPV^33^ . Apesar da constatação de que se trata de um câncer em declínio em vários países, tendências recentes de aumento da incidência entre mulheres jovens, mais marcadas em países de alta renda, têm sido observadas^34 , 35^ . Para coortes de nascimento a partir de 1940 ou 1950 observou-se um aumento da incidência em países da Europa, Japão e estabilidade nos Estados Unidos^3^ . Da mesma forma foi descrito na Europa Central, no Leste Europeu e na Ásia Central^33^ . Esse aumento tem suscitado debate em torno da necessidade de se rever e implementar estratégias de rastreamento mais efetivas.

Nas idades mais jovens, as taxas de mortalidade por câncer são mais baixas do que as taxas nas idades mais avançadas, sendo, portanto, mais propensas a flutuações devido ao menor número de mortes. Neste estudo, a tendência de aumento da mortalidade em mulheres jovens foi verificada a partir de variações percentuais anuais positivas e estatisticamente significativas. Por esse motivo, consideramos relevante mostrar essa situação que vem sendo relatada em outros países. No Brasil, esse achado requer atenção aos indicadores de cobertura do rastreamento nesse grupo etário específico, bem como aos indicadores de acesso ao diagnóstico e tratamento, já que nessas idades são esperados menor número de mortes por câncer de colo do útero. Conhecer a prevalência da infecção por HPV ao longo do tempo e se a doença tem acometido essas mulheres mais precocemente trariam contribuições importantes para o direcionamento de ações específicas em todos os níveis de atenção à saúde.

As coberturas de Papanicolaou autorreferidas para todas as mulheres na faixa etária alvo foram de 78,8% no país^36^ e de 80% nas capitais^37^ , proporções que podem ser consideradas elevadas. Por serem baseadas em informações autorrelatadas por mulheres entrevistadas em inquéritos populacionais, elas podem não refletir a real cobertura do rastreamento. Ao mesmo tempo, observa-se que as taxas de incidência e mortalidade por câncer de colo do útero permanecem altas se comparadas a de outros países^2^ , com grande disparidade entre regiões^38^ . Nesse aspecto, vale ressaltar que a cobertura real é certamente menor do que as referidas em inquéritos populacionais.

A partir das bases de dados do SUS – SISCOLO, SISCAN e SIA são observadas coberturas mais baixas. Recentemente, foi identificada queda no número de mulheres que realizaram exame citopatológico pela primeira vez no SISCOLO, chegando a 41% entre os anos de 2012 e 2013^39^ . Além disso, a oferta de exames no SUS para confirmação diagnóstica é deficitária, o que prejudica o seguimento de mulheres rastreadas^40^ .

No Sudeste, especialmente em São Paulo, a cobertura do rastreamento no SUS tende a ter níveis mais altos se comparada aos demais estados do país. Em 2009–2020, as quedas nos percentuais de cobertura do exame de rastreamento foram observadas em todos os estados e faixas etárias, indicando que o acesso ao exame no SUS vem sendo reduzido. O nível mais baixo de cobertura ocorreu em 2020, o que pode ser explicado pela pandemia de covid-19.

Vale et al.^41^ encontraram para Campinas, município localizado no estado de São Paulo, redução significativa na proporção de exames realizados fora do grupo etário alvo do rastreamento entre 2010 e 2016, especialmente, em mulheres com idades inferiores a 25 anos. Segundo os autores, o melhor alinhamento das práticas locais às diretrizes nacionais poderia explicar as quedas observadas nos percentuais de cobertura nos últimos anos. Além de redução no excesso de exames, Vale et al.^41^ encontraram aumentos nas proporções de exames realizados entre 25 e 64 anos, fato não observado neste estudo. Nos dados apresentados para a região Sudeste, as reduções observadas desde 2009 no grupo de 65 anos ou mais também foram verificadas nas idades de 25–64 anos. Esse fato sugere que existem outros fatores relacionados à queda da cobertura de exames na região Sudeste, além daqueles apontados no estudo de Campinas^41^ .

Além da queda de cobertura, há problemas no seguimento dos resultados alterados. Estudo que analisou a qualidade do seguimento de mulheres rastreadas no estado de São Paulo, a partir do relacionamento das bases de dados do SISCAN, SIA e Sistema de Internações Hospitalares (SIH), mostrou que para 35,2% das mulheres com citologias alteradas não foram encontrados dados sobre o diagnóstico nos sistemas de informação^11^ . Além disso, identificou tempo mediano maior que seis meses entre o exame alterado e o diagnóstico e quase três meses entre o diagnóstico e o início do tratamento. Esses tempos prolongados se associaram a piores condições de assistência nas regionais de saúde do estado. Reforçando esses achados, outro estudo conduzido também em São Paulo concluiu que o acesso à colposcopia é limitado no estado, prejudicando o diagnóstico e, consequentemente, o tratamento^42^ .

Atrasos no diagnóstico levam a diagnósticos em fases mais avançadas. Nesse aspecto, as ações de rastreamento para o câncer de colo uterino têm papel importante, não apenas na redução da incidência da doença, mas também na redução da mortalidade por propiciarem diagnóstico precoce.

Estudo conduzido no Brasil, a partir de dados de registros hospitalares de câncer, mostrou que o diagnóstico do câncer de colo do útero ocorreu tardiamente (estádios III–IV), em 53,5% dos casos em 2012^43^ . Em São Paulo, dados hospitalares de tumores invasivos de colo uterino^44^ , diagnosticados em 2017, apontaram que 39,2% dos casos em mulheres de 25–39 anos foram diagnosticados em estádios III–IV. Nas faixas etárias de 40–64 e 65 anos ou mais, as respectivas proporções foram 51,7% e 62,3%. Esses dados sugerem um cenário preocupante, considerando que o programa nacional de controle do câncer de colo do útero está implantado em todo o país desde o final da década de 1990. Melhorias na detecção precoce desse câncer teriam importante efeito na redução da mortalidade no país.

A descoberta do papel causal do HPV^1^ traz como consequência a necessidade de reformular a prevenção primária e secundária do câncer de colo do útero^6^ . A introdução do teste de HPV pode otimizar e tornar o rastreamento mais efetivo, especialmente em países de baixa renda^45^ . Dados de grandes estudos randomizados evidenciaram que a proteção contra o carcinoma invasivo – com rastreamento baseado nos testes de HPV a partir dos 30 anos de idade e com intervalo de 5 anos – é 60–70% maior se comparado à citologia oncótica^7^ .

Existem, contudo, questões que merecem investigação para tomada de decisões sobre medidas estratégicas e custo-efetivas que devem ser implementadas nos programas de controle do câncer de colo do útero. Entre elas, definição dos intervalos do rastreamento, formas para motivar a adesão de mulheres e organização dos serviços contemplando desde o acesso ao rastreamento até o aumento da infraestrutura para diagnóstico e tratamento^46^ . Mesmo com a disponibilidade do teste de HPV como exame recomendado para rastreamento, desafios inerentes à organização do programa continuarão existindo. Mais do que o teste de rastreio, o emprego da abordagem mais adequada para organizar todos os componentes, incluindo os aspectos de qualidade, representam fatores determinantes para o sucesso de um programa de rastreamento^8^ .

As análises de tendência de mortalidade por câncer de colo do útero são frequentemente comprometidas por imprecisões no preenchimento da causa básica de morte, pelo fato de uma parcela ser registrada como câncer de útero de porção não especificada, o que não permite o conhecimento da verdadeira origem anatômica do tumor (colo ou corpo do útero). Para lidar com essa limitação, foi empregada técnica de correção dos óbitos por câncer de colo do útero originalmente registrados no SIM, possibilitando análises mais realistas. É importante destacar que em 2020 a correção por causas mal definidas foi baixa em todas as regiões (variou de 1,96% no Centro-Oeste a 4,21% no Norte). No entanto, a redistribuição por útero porção não especificada levou a um aumento de 28,57% na região Sudeste, percentual maior se comparado às demais regiões.

Após significativo progresso na acurácia da informação acerca do óbito no período 1980–2020 e décadas de declínio na mortalidade pelo câncer de colo do útero, tendências recentes de estabilidade no Brasil e de aumento no estado de São Paulo apontam para a necessidade de reorganização do atual programa de rastreamento para se alcançar melhorias na cobertura e na qualidade em todas as suas etapas – rastreio, diagnóstico e tratamento. A imunização contra o HPV trará resultados positivos a longo prazo, mas sua implementação não minimiza o papel da prevenção secundária; pelo contrário, reforça a necessidade imediata de planejamento para implementação a médio prazo de um exame de rastreamento mais custo-efetivo e de maior sensibilidade.

Um programa de rastreamento organizado permitirá alcançar ativamente as mulheres da faixa etária alvo e, em especial, as de 25–39 anos, em que se observou aumento acentuado da mortalidade por câncer de colo do útero no país. São mulheres em plena atividade sexual e, também, claramente integradas à população economicamente ativa, portanto, com maiores dificuldades de adesão ao rastreamento.

Somente com abordagem ampla, que inclua alta cobertura, qualidade dos exames e seguimento em toda a linha de cuidado, serão alcançadas maiores reduções não só da mortalidade, mas também da incidência. Assim, será garantido o alinhamento do Brasil à estratégia global de eliminação do câncer de colo do útero como problema de saúde pública^47^ .
